# Special Issue: Artificial Intelligence and Smart Sensor-Based Industrial Advanced Technology

**DOI:** 10.3390/s24227391

**Published:** 2024-11-20

**Authors:** Luyu Jia, Bairong Sun, Weilin Tan, Shurong Zhang, Bin Zhang, Jianxiong Zhu

**Affiliations:** 1 Sustainable Development Research Group, Chongqing University of Technology, Chongqing 400054, China; jialy@cqut.edu.cn; 2 School of Mechanical Engineering, Southeast University, Nanjing 211189, China; 220234882@seu.edu.cn; 3 School of Mechanical Engineering, Southeast University, Suzhou Campus, Suzhou 215125, China; 4 Xiaomi Smart Home Appliances (Wuhan) Co., Ltd., Nanjing 210036, China; tanweilin@xiaomi.com (W.T.); zhangshurong@xiaomi.com (S.Z.)

With the rapid growth of smart sensors and industrial data, artificial intelligence (AI) technology (such as machine learning, machine vision, multi-sensor fusion, cloud computing, edge computing, digital twins, etc.) now exhibits the potential to significantly enhance manufacturing processes and industries [1]. In recent years, a large number of research organizations have focused on intelligent process and manufacturing technologies, resulting in extensive innovation and advancements in the sector [2,3,4]. Intelligent manufacturing technologies, which include smart sensor deployment [5], IoT sensor nodes [6], virtual reality [7], fussy systems [8], etc., may be modified to create process control systems and technologies for the prediction of quality prediction and the detection of defects [9,10,11]. Multi-sensor-based intelligent process control systems may eventually become a reality thanks to cutting-edge machine learning algorithms and enhanced computed performance [12], offering a wide range of exciting new applications in the coming era, including robotics [13], smart factories [14], smart homes [15], and decision support systems [16].

This Special Issue features 11 research and review articles that present recent advances in multi-sensor fusion; machine vision technologies; digital twin technologies; human–machine interfaces; IoT; machine learning; big data; and other applications [17,18,19]. This ought to provide readers with an overview of the difficulties, prospects, and trends associated with the design, manufacturing, characterization, integration, and use of sophisticated industrial process systems and artificial intelligence.

In this Special Issue, six research articles explore novel approaches to defect detection in industrial systems. Zhang et al. (Contribution 1) introduce ODNet, a real-time network for the few-shot classification f strip steel surface defects, utilizing orthogonal decomposition to enhance precision and generalization in industrial settings. Using cellphones for real-time monitoring, Kim et al. (Contribution 2) present a method that can be used to identify road faults, exceeding traditional models in terms of processing speed and accuracy. Say et al. (Contribution 3) utilize convolutional neural network (CNN) techniques for the automatic detection of welding defects in X-ray images. The effectiveness of the proposed method is confirmed by testing its performance during the processing of an industrial dataset, which can be used in contemporary solutions for the automated detection and categorization of welding defects. Then, Luo et al. (Contribution 4) develop an improved YOLOv5-based object detection system for coal mine conveyor belts, emphasizing both speed and accuracy. Using one-class classification (OCC) models, Lee et al. (Contribution 5) investigate two-stream networks for the identification of defects in unbalanced datasets. This approach prevents the decision border from collapsing to the training dataset and produces a suitable decision boundary. To minimize the number of measurement errors caused by a loss of contact between the odometer wheel and the pipe, Freitas et al. (Contribution 6) concentrate on using neural networks in the oil and gas sector to forecast the speed of pipeline inspection gauges (PIGs).

Regarding the innovations and applications of vision algorithms, Hua et al. (Contribution 7) present a high-precision hand–eye coordination method based on convex relaxation optimization, improving the accuracy, noise resistance, and stability of switch operations for electric control cabinets. Meanwhile, Yang et al. (Contribution 8) combine global and local feature fusion techniques in order to perform vehicle re-identification in intelligent transportation, significantly improving the model’s generalization ability. Xu et al. (Contribution 9) apply deep reinforcement learning to control multivariable coupled systems, enhancing stability and precision. To examine sleep patterns without making contact, Moshayedi et al. (Contribution 10) develop a vision-based system to analyze the emotional effects of news on sleep patterns, potentially aiding in the diagnosis and study of sleep-related diseases. Zou et al. (Contribution 11) propose a method based on generative adversarial networks (GANs) for the super-resolution reconstruction of coal photomicrographs. The proposed method captures long-range feature correlations across multiple scales, generating more explicit and realistic results.

We hope that these articles not only provide valuable insights into the field, but also inspire new ideas and innovations in related research and applications, driving the development and practice of artificial intelligence and smart sensor-based industrial advanced technology; this will enable the industry to achieve greater breakthroughs in the future.
